# Declared Intention (Not) to Be Vaccinated against COVID-19, and Actual Behavior—The Longitudinal Study in the Polish Sample

**DOI:** 10.3390/vaccines10020147

**Published:** 2022-01-20

**Authors:** Jozef Maciuszek, Mateusz Polak, Katarzyna Stasiuk

**Affiliations:** Institute of Applied Psychology, Faculty of Management and Social Communication, Jagiellonian University in Krakow, 31-007 Krakow, Poland; mateusz.polak@uj.edu.pl (M.P.); katarzyna.stasiuk@uj.edu.pl (K.S.)

**Keywords:** COVID-19 vaccination, intention to vaccinate, vaccination uptake, theory of reasoned action, beliefs about vaccination

## Abstract

AIMS: The aim of the study was to investigate the relation between declared intention to get/not get vaccinated against COVID-19, prior to the start of the global vaccination program, and actual vaccine uptake. Moreover, reasons for getting vaccinated or rejecting it were measured along with declared intent and behavior. METHODS: Within a longitudinal design, a representative sample of 918 Polish people was surveyed in February 2021 and August 2021. In February 2021, participants were asked about their intention to get vaccinated against COVID-19 and the reasons behind it. In August 2021, the same group was asked about having been vaccinated, along with the reasons. RESULTS: A significant pro-vaccine shift from declared intent to behavior was observed, with many participants turning away from being anti-vaccine or undecided and getting vaccinated. Significant correlations with attitudes toward general mandatory vaccination of children were found. Increased support for anti-vaccine arguments was seen over time in the unvaccinated sample, and decreased support for pro-vaccine arguments was seen in the vaccinated sample. Several key arguments for and against vaccination were identified. CONCLUSIONS: Declared attitude toward COVID-19 vaccination is not fully consistent with vaccination behavior. Pro-vaccine changes in attitudes of previously anti-vaccine and undecided individuals indicate that these groups may be influenced to potentially accept the COVID-19 vaccination over time.

## 1. Introduction

In the beginning of 2020, the COVID-19 outbreak affected the entire world’s functioning and has become a global pandemic, giving rise to a serious health threat. On 30 January 2020, COVID-19 was declared a Public Health Emergency of International Concern (PHEIC) with an official death toll of 171. Poland, where we conducted our study, suffered relatively low rates of infections during the first wave of the pandemic, but has subsequently been hit hard by the second, with the number of cases growing exponentially during October 2020. On 15 December 2020, the National Vaccination Program for the Prevention of Coronavirus Disease 2019 (COVID-19) was adopted in Poland. The Polish Prime Minister and Minister of Health, while announcing details of plans to introduce the COVID-19 vaccine, expressed optimism that while vaccination would remain voluntary, up to 80% of the population would opt for it [1].

Announcements to start mass vaccination from February 2021 have raised hopes of rapid containment of the pandemic, but also doubts about the willingness of the population to be vaccinated to an extent necessary to achieve herd immunity. These concerns stemmed both from growing general vaccine hesitancy in Poland in the last decade before the pandemic, and from polls measuring the intention to be vaccinated specifically against COVID-19. In the decade before the outbreak of the pandemic, vaccine skepticism increased, anti-vaccine movements became more active, and vaccination rates declined in some countries (see, e.g., [2]). In Poland, the number of evasions of mandatory child vaccinations rose from 3437 in 2010 to 48,609 in 2019. Additionally, public opinion polls conducted before the start of the COVID-19 vaccination program showed worryingly low interest in receiving the COVID-19 vaccine. In one of the polls conducted in November 2020, only 36 percent of respondents said they would choose to be vaccinated, while 47 percent said they would not, and 17 percent were undecided [3]. During the course of the pandemic, many studies were conducted in different countries to examine the number of people declaring willingness to receive the vaccine against SARS-CoV-2, e.g., [4], the predictors of intention to vaccinate (e.g., knowledge about vaccination, or personal experience with COVID-19, [2]), and the differences in attitudes toward vaccination depending on demographic factors, e.g., [5]. Our study adds to the existing literature firstly because it aimed to investigate not only the intention to vaccinate against COVID-19 (right after the start of the vaccination campaign), but also the actual behavior (vaccination or not) of the same group of people six months later. Moreover, we examined not only the reasons for the decision not to vaccinate (which has been examined in many studies, e.g., [6]) but also how those who wanted to receive the vaccination (in the first measurement) or had been vaccinated (in the second measurement) justified their choice (which, to our knowledge, has been less frequently studied).

### 1.1. What Drives Vaccine Skepticism?

Prior to the outbreak of the current pandemic, various factors of vaccine skepticism have been pointed out. Of most interest has been the role of individual differences in the formation of vaccine skepticism (religious orthodoxy, conspiracy thinking, hierarchical worldview, among others) [7,8]. Vaccine skepticism may be shaped unintentionally by the messages and attitudes of opinion-based groups, such as health professionals [9] and journalists [10]. Organized anti-vaccine movements spreading their arguments through websites, which are growing in popularity, are also an important source of information for vaccine skeptics. People who refuse vaccination justify their position most frequently by their fear of the side effects of vaccination, their belief that vaccinations do not in fact protect against disease, and their distrust of pharmaceutical companies [11,12,13,14].

### 1.2. Acceptance or Rejection of Vaccinations as a Consequence of Conscious Intention-Own Research

The starting point of our research plan was the theory of reasoned action by I. Ajzen [15]. Theory of reasoned action assumes that an attitude influences an action through a conscious intention, which depends on the attitude towards this action and the subjective norm. According to this theory, behavior is a direct consequence of a person’s conscious intention. The content of the intention is determined, first, by the attitude towards the action (which depends on the conviction of what results the action leads to and on the evaluation of those results) and second, by the conviction of what behavior is expected, required, or dominant in a given social environment. Of course, attitude does not always influence behavior through conscious intentions (see [16]). Intentions do, however, play a large role in infrequently performed, non-habitual behaviors (including vaccination).

The theory of reasoned action can be applied to vaccine behavior. Vaccine acceptance or rejection is associated with conscious, deliberate intention (vaccination is not a frequent or habitual behavior) and depends to a large extent on one’s own attitude toward vaccination, which is shaped by one’s opinions about the effects and consequences of vaccination, its benefits and risks. The compliance of attitudes to actual behavior can be strengthened or weakened by the behaviors and attitudes of the social environment, especially important people—following the majority or yielding to social pressure can lead to behaviors that are inconsistent with one’s own attitudes toward vaccination.

The assumptions of the theory of reasoned action became the basis for the design of the present study and for predicting and explaining people’s behavior during the universal vaccination program against COVID-19. We planned to measure attitudes toward general vaccination (by means of a question about attitudes toward the mandatory traditional vaccination of children) and then the intention to be vaccinated against COVID-19 when possible (declaration of acceptance vs. rejection of vaccination vs. no decision). Crucially, we then measured whether the declared intent to (not) get vaccinated against COVID-19 was reflected in actual behavior—whether pro-vaccine individuals would actually get vaccinated, whether anti-vaccine individuals would actually not take the vaccine, and what action would the undecided ones take. In reference to the theory of reasoned action, we were also interested in (a) what role the individual motives and reasons given play in justifying their intention (first measurement) and causing their behavior (second measurement), (b) which of these reasons will have the greatest importance in the expression of one’s intention of (not) getting vaccinated, and in the actual behavior (vaccination or rejection), and (c) whether and to what extent the importance rating of each reason will change between the first and second measurements.

## 2. Methods

### 2.1. Participants and the Study Design

A representative sample of the Polish general population (*n* = 918) took part in both measurements of the longitudinal study. Given the population of adult Polish citizens (estimated at 26 million), 95% CIs and an α = 0.05, the minimum sample size would be *n* = 385. We put efforts to make our sample size larger than that. The study was run by the Ariadna Nationwide Research Panel, a Polish counterpart of mTurk—a company specialized in polling of large samples for the purpose of research. The panel enables random selection of a sample from among 300,000 registered and verified persons. The sociodemographic profile of the persons registered on the panel corresponds with the profile of Polish internet users. Additionally, Ariadna has been awarded certificates issued by recognized organizations associated with social research companies (including ESOMAR). For participation in the survey, respondents received credit points that they could exchange for gifts. A random quota sampling method was used, based on sex (2 subgroups), age (5 subgroups), place of residence (5 subgroups), and education (6 subgroups), each demographic criterion controlled to be representative of the Polish general population—giving a total of 50 weighted cells. Weights were calculated based on these four demographic criteria, and participants were drawn randomly from cells to fit demographic quotas. The first measurement of the study (*n*= 1391, hereafter called Time 1) was run in February 2021, before the start of the vaccination program for regular citizens. We reached out to the same group of respondents in August 2021 (hereafter called Time 2), when vaccines were readily available and nearly all willing citizens of Poland had the opportunity to get vaccinated with at least one dose. Reaching the same sample for a second time was possible as Ariadna uses unique identifiers for their respondents and there is a possibility to reach out to particular respondents inviting them for a given survey. In the second measurement we were able to reach most of the participants from that took part in the first measurement, and we finally conducted analyses on the 918 individuals who participated in both measurements.

The final sample was 61% women and 39% men; 9% were aged 18–24, 26%—between 25 and 34, 23% were aged between 35 and 44, 18% were aged between 45 and 54, and 23% were aged 55 and more; 10% had primary or vocational education, 42% had secondary or postsecondary education, and 48% had a bachelor’s degree. Moreover, 34% of participants live in the rural/small town (under 20 k residents), 24% in medium town (20 k–99 k residents) and 42% in city or large city (100 k–over 500 k residents).

### 2.2. Procedure

Questionnaires were presented online on the Ariadna survey platform. The questionnaire was divided into three parts and required on average 10 min to be completed. The first part was demographic data, including age, gender, education, place of residence. The second part of the questionnaire included questions about attitudes toward mandatory vaccination of children against common diseases (“Do you support mandatory vaccinations for children?”) and support for the introduction of mandatory vaccination for COVID-19 (“Do you think COVID-19 vaccination should be mandatory?”) with an 11-point Likert scale from 0—*definitely no*—to 10—*definitely yes*. The third part was a declaration of willingness to be vaccinated against COVID-19 (at Time 1) or stating whether the subject had actually been vaccinated (at Time 2). At Time 1, respondents were asked “Would you vaccinate against COVID-19 as part of a national vaccination program?” with 5 possible responses: 1—*definitely no*, 2—*no*, 3—*I have not decided yet*, 4—*yes*, and 5—*definitely yes*. At Time 2, respondents were asked “Did you get vaccinated against COVID-19?” with 8 possible responses: 1—*no, and I’m not going to get vaccinated*, 2—*no, and I don’t know if I will get vaccinated*, 3—*yes, with two doses*, 4—*yes, with a single-dose vaccine*, 5—*yes, with one dose and I intend to take the second one*, 6—*yes, with one dose, but I do not intend to take the second,* 7—*yes, with one dose, but I’m not sure if I take the second one,* 8—*not yet, but I plan to get vaccinated.*

In the third part of the questionnaire, respondents were asked about the reasons for their COVID-19 vaccination intention/behavior. Respondents who declared that they did not intend to get vaccinated (Time 1) or did not get vaccinated (Time 2), and those who were undecided were asked to respond to a list of eight possible reasons for their decision using an 11-point Likert scale from 0—*I definitely do not agree with this reason*—to 10—*I definitely agree with this reason.* The reasons presented to this group were as follows: “I do not believe that the coronavirus actually exists”; “I do not believe that the coronavirus is dangerous”; “I am afraid of the side effects of the vaccine”; “I have already had COVID-19 and am not afraid of getting it again”; “I do not believe in the sincerity of vaccine companies”; “There is a small chance that I will get coronavirus”; “Too few clinical studies have been done”; “I am not sure if the vaccine is safe or effective because it was not developed long enough”. Post hoc Cronbach’s alphas for this measure were α = 0.796 at Time 1 and α = 0.738 at Time 2. The respondents who declared that they intend to get vaccinated (Time 1) or got vaccinated (Time 2) were also asked to respond to a list of eight possible reasons for their decision using a 10 point Likert scale from 0—*I definitely do not agree with this reason*—to 10—*I definitely agree with this reason.* The reasons presented to this group were: “I will protect myself from the danger of contracting the virus”; “I will increase the safety of family members, friends and acquaintances, and strangers”; “By getting vaccinated I will break the chain of infections and help fight the global pandemic”; “Coronavirus vaccines are voluntary and free”; “I will gain the inner peace that comes from keeping myself and my loved ones safe”; “The vaccines are safe, their approval is dependent on the decisions of credible medical agencies”; “Vaccines are the most effective method to protect against contracting various diseases, mankind has been successfully using this achievement of civilization for several hundred years”; “I will get back to my normal life faster with the release of many restrictions in my country and while traveling abroad”. Post hoc Cronbach’s alphas for this measure were α = 0.912 at Time 1 and α = 0.955 at Time 2.

## 3. Results

### 3.1. Attitude toward Mandatory Vaccination of Children vs. Attitude toward Mandatory Vaccination against COVID-19

We found a significant, moderate positive correlation between participants’ attitudes toward mandatory vaccination of children a Time 1 and Time 2 (Kendall’s τ = 0.573, *p* < 0.001). A paired samples t-test showed that these attitudes were not significantly different between Time 1 and Time 2 (M = 8.17, SD = 2.66 vs. M = 8.03, SD = 2.80; t(917) = 1.731, *p* = 0.084).

Attitude toward mandatory vaccination of children was positively correlated with attitude toward mandatory vaccination against COVID-19 (τ = 0.326 for Time 1, τ = 0.358 for Time 2, both *p* < 0.001).

Moreover, there was a moderate positive correlation between support for mandatory vaccination against COVID-19 at Time 1 and Time 2 (τ = 0.60, *p* < 0.001). A paired-samples t-test indicated an increase in support toward mandatory vaccination against COVID-19 between Time 1 (M = 4.61, SD = 3.80) and Time 2 (M = 4.97, SD = 4.09; t(917) = 3.637, *p* < 0.001, Cohen’s d = 0.24). There were significant differences in support for mandatory vaccination against COVID-19 between anti-vaccine, undecided and pro-vaccine individuals at Time 1 (F(2915) = 519.589, *p* < 0.001, η^2^ = 0.532), the highest support found in pro-vaccine (M = 7.55, SD = 2.84), lowest in anti-vaccine (M = 0.92, SD = 1.98) and moderate support in the undecided group (M = 3.36, SD = 2.67; all *p* < 0.001 Bonferroni corrected). Similar differences were seen at Time 2 (F(2872) = 420.083, *p* < 0.001, η^2^ = 0.491) between vaccinated (M = 7.09, SD = 3.27), undecided (M = 1.55, SD = 2.57) and unvaccinated individuals (M = 0.61, SD = 2.57; all Bonferroni *p* < 0.02)

### 3.2. Declared Intent of Vaccinating against COVID-19 vs. Actual Vaccination

We measured declared intent of COVID-19 vaccination at Time 1 (Will you get vaccinated against COVID-19 when a vaccine is available?) and action taken at Time 2 (Did you get vaccinated against COVID-19). At Time 1, we identified 225 anti-vaccine, 404 pro-vaccine and 289 undecided individuals. At Time 2, the same sample consisted of 162 anti-vaccine, 567 vaccinated and 137 undecided individuals (the remaining 43 were excluded from the study due to providing the following ambiguous answers: *Not yet but I am going to get vaccinated*, *I took one dose but don’t know if I will take the second one* and *I took one dose but I don’t want to take the second one*—these answers did not allow putting them into a clear category).

McNemar-Bowker test indicated a significant shift in participants’ responses between Time 1 and Time 2 (χ^2^ (3, *n* = 875) = 161.612, *p* < 0.001, Cramer’s V = 0.519). Results indicated that the shift was toward vaccination. In total, 43 of the previously anti-vaccine individuals became undecided, and 48 got vaccinated. In total, 149 of the previously undecided group got vaccinated and 30 became anti-vaccine. Crucially, only fourteen of the previously pro-vaccine group did not get vaccinated (six becoming anti-vaccine and eight undecided) Results are presented in Table 1.

### 3.3. Reasons to Get Vaccinated or Not, and Their Association with Intended and Actual Vaccination

We then analyzed the declared reasons for getting or not getting vaccinated at Time 1 and Time 2. We only asked pro-vaccine individuals about why they want to vaccinate against COVID-19, and only asked anti-vaccine and undecided individuals about why they do not want to vaccinate. This meant that we could run these analyses only on the consistently pro-vaccine and consistently anti-vaccine/undecided samples).

At Time 1, the most supported arguments against vaccination were “Afraid of side effects” and “Too little research conducted” (similar level with Bonferroni *p* > 0.999), shortly followed by “Not sure if vaccine is safe and effective” (*p* = 0.014) and then “Don’t believe companies producing the vaccines are honest” (lower with *p* < 0.001), the remaining arguments much less supported (all *p* < 0.001). A similar structure was seen at Time 2, the most supported arguments being “Afraid of side effects”, “Not sure if vaccine is safe and effective”, and “Too little clinical research conducted” (similar with Bonferroni *p* > 0.999), followed by “Don’t believe companies producing the vaccine are honest” (lower with *p* < 0.001) and then the remaining arguments. Descriptive statistics can be found in Table 2.

**Figure 1:**
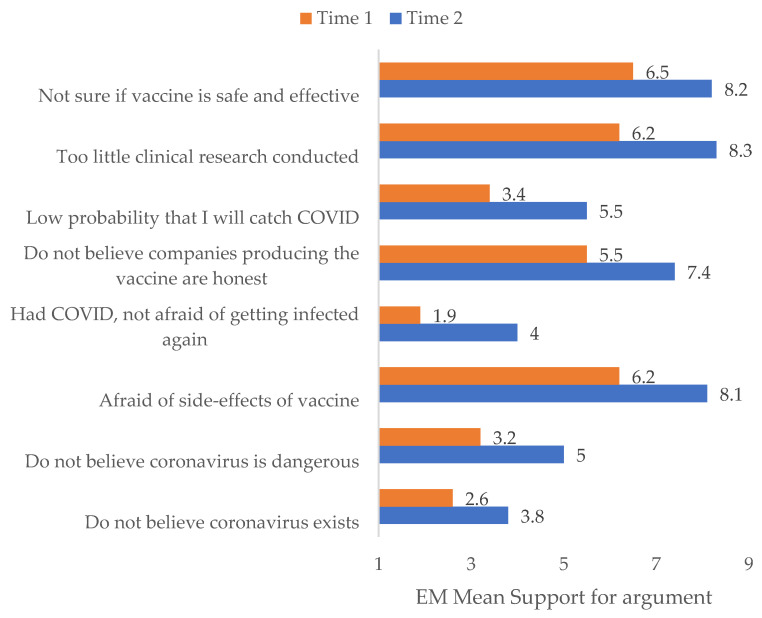
Reasons to not get vaccinated against COVID-19 between Time 1 and Time 2.

We used a repeated measures ANOVA to compare the overall declared reasons to not vaccinate between Time 1 and Time 2. It turned out that support for all the reasons to not get vaccinated was significantly higher at Time 2 than at Time 1 in the anti-vaccine and undecided samples (F(1284) = 334.410, *p* < 0.001 η^2^_p_ = 0.541); all pairwise comparisons *p* < 0.001 (Bonferroni corrected). Results are presented in Table 2 and Figure 1.

**Figure 2:**
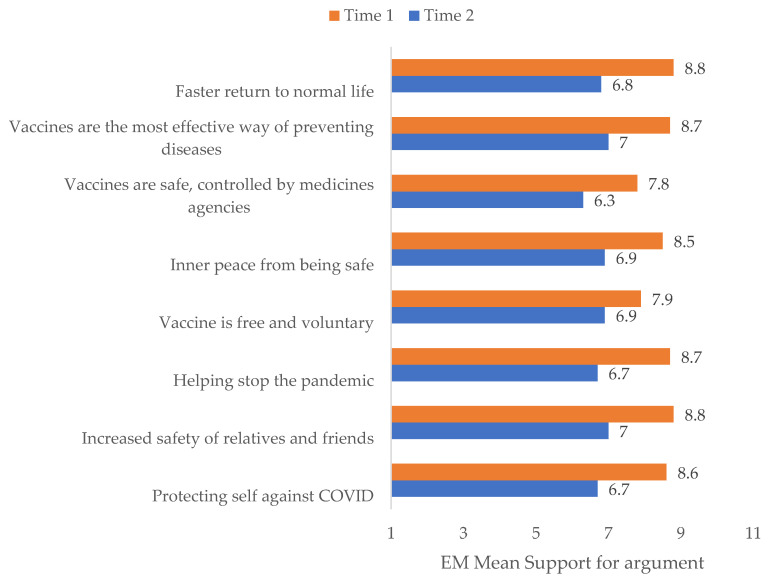
Reasons to get vaccinated against COVID-19 between Time 1 and Time 2.

Conversely, it turned out that support for all the reasons to get vaccinated was significantly lower at Time 2 than at Time 1 in the pro-vaccine sample (F(1388) = 573.765, *p* < 0.001, η^2^_p_ = 0.597); all pairwise comparisons *p* < 0.001 (Bonferroni corrected). Results are presented in Table 3 and Figure 2.

The most important reasons to get vaccinated against COVID-19 at Time 1 were Increased safety of relatives and friends, “Helping stop the pandemic”, “Vaccines are the most effective way of preventing diseases”, and “Faster return to normal life”, all similar with Bonferroni *p* > 0.70. At Time 2 there was much lower variation between support for reasons, the most supported being “Vaccines are the most effective way of preventing diseases” (most supported with all Bonferroni *p* < 0.001), shortly followed by “Increased safety of relatives and friends”, “Helping stop the pandemic”, “Vaccine is free and voluntary”, “Inner peace from being safe”, and “Faster return to normal life” (the five arguments were similarly supported with *p* > 0.999). Descriptive statistics are presented in Table 3.

The next analysis was focused on identifying the differences between those who consistently remained anti-vaccine or hesitant, and those who changed their attitude between Time 1 and Time 2. We were unable to perform these analyses on the (in)consistently pro-vaccine sample, since only 14 out of the 393 pro-vaccine individuals shifted to being anti-vaccine or undecided.

For the initially (Time 1) anti-vaccine group, we found significant differences in support for the Time 1 reasons to not vaccinate between those who remained anti-vaccine, those who shifted toward undecided and those who got vaccinated against COVID-19 (F(2214) = 3.593, *p* = 0.029, η^2^_p_ = 0.032). Pairwise comparisons (Bonferroni corrected) indicated that there were significant differences between consistently anti-vaccine and those who shifted from anti-vaccine to undecided in “Don’t believe companies producing the vaccine are honest” (M = 6.37, SE = 0.23 vs. M = 5.14, SE = 0.39, *p* = 0.022). All other pairwise comparisons turned out to be nonsignificant.

For the initially undecided group, we found no differences in support for the Time 1 reasons to not vaccinate between those who remained undecided, those who shifted to being anti-vaccine and those who got vaccinated (F(2262) = 0.186, *p* = 0.830, η^2^_p_ = 0.001).

For the finally (Time 2) anti-vaccine group, we found no significant differences in support for Time 2 reasons to not get vaccinated between those who remained anti-vaccine, those who shifted from undecided to anti-vaccine, and those who shifted from pro-vaccine to anti-vaccine (F(2159) = 0.344, *p* = 0.710, η^2^_p_ = 0.004).

For the finally undecided group, we found no significant differences in support for Time 2 reasons to not get vaccinated between those who shifted from anti-vaccine to undecided, those who were previously undecided, and those who shifted from pro-vaccine to undecided (F(2134) = 1.337, *p* = 0.266 η^2^_p_ = 0.020).

**Figure 3:**
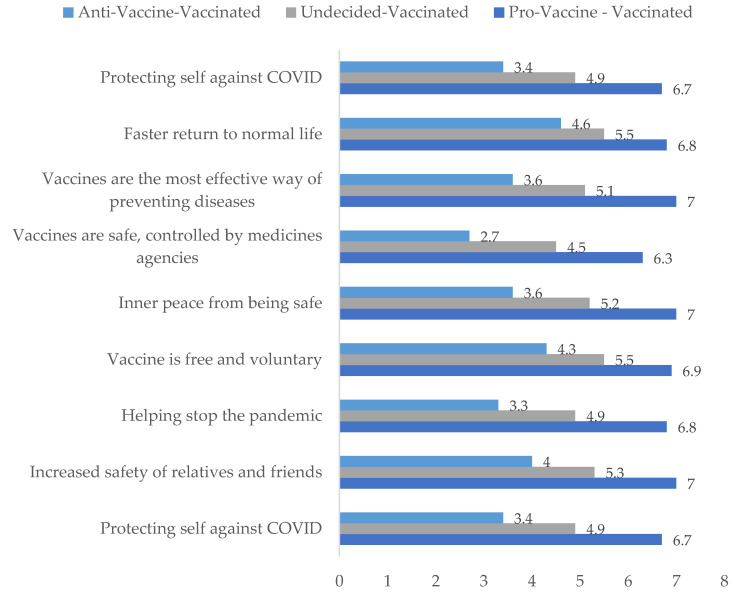
Reasons to get vaccinated against COVID-19 between previously anti-vaccine, undecided and pro-vaccine individuals who got vaccinated.

We did, however, find significant differences in support for Time 2 Reasons to get vaccinated between the consistently pro-vaccine, those who shifted from undecided to vaccinated, and those who shifted from anti-vaccine to vaccinated (F(2573) = 119.351, *p* < 0.001, η^2^_p_ = 0.294). It turned out that despite all getting vaccinated against COVID-19, these three groups exhibited different levels of support for all the reasons to vaccinate (all pairwise comparisons *p* < 0.001, Bonferroni corrected). The consistently pro-vaccine individuals demonstrated the highest support, the anti-vaccine turned vaccinated exhibited the least support, and the undecided turned vaccinated were somewhere between these two groups. Results are presented in Table 4 and Figure 3.

### 3.4. Attitudes toward Mandatory Vaccination of Children and COVID-19 Vaccination Support

The next set of analyses concerned participants’ attitude toward mandatory vaccination of children (at Time 1 and Time 2), and mandatory vaccination against COVID-19 (Time 2).

We found significant differences in attitudes toward mandatory vaccination of children between declared anti-vaccine, pro-vaccine and undecided groups (at Time 1)—F(2915) = 120.327, *p* < 0.001, η^2^ = 0.208. Pairwise comparisons (Bonferroni) indicated that the highest support was in the pro-vaccine group (M = 9.41, SD = 1.27), then the undecided group (M = 7.81, SD = 2.44) and the lowest in the anti-vaccine group (M = 6.41, SD = 3.51, all *p* < 0.001).

Similarly, we found significant differences in attitudes toward mandatory vaccination of children (at Time 2) between those who got vaccinated, those anti-vaccine and those undecided (F(2872) = 92.322, *p* < 0.001, η^2^ = 0.175). The highest support was in the pro-vaccine group (M = 8.82, SD = 2.84), then in the undecided group (M = 6.86, SD = 3.20) and then in the anti-vaccine group (M = 5.97, SD = 3.61; all Bonferroni *p* < 0.01).

There was a significant correlation between declared support for vaccination of children at Time 1 and Time 2 (Kendall’s τ = 0.573, *p* < 0.001). Moreover, a mixed ANOVA (Time x Declaration/Action of getting vaccinated against COVID) indicated significant general reduction in support toward children’s mandatory vaccination between Time 1 and Time 2 (M = 7.80, SE = 0.167 vs. M = 7.29, SE = 0.176; F(1866) = 9.674, *p* = 0.002, η^2^_p_ = 0.009) as well as a significant interaction between Time and Declaration/Action (F(8866) = 7.448, *p* = 0.006, η^2^_p_ = 0.021). Pairwise comparisons (Bonferroni corrected) indicated that there was a significant reduction in support toward mandatory vaccination in Undecided who became Unvaccinated against COVID-19 (M = 7.76, SE = 0.434 vs. M = 6.30, SE = 0.459, *p* < 0.001), as well as those who remained consistently undecided between Time 1 and Time 2 (M = 7.61, SE = 0.256 vs. M = 6.90, SE = 0.271, *p* = 0.005). There were no significant differences in this regard in any other group.

## 4. Discussion

In recent years, before the outbreak of the pandemic, growing vaccine skepticism and declining vaccination rates have been observed in many countries. At the time of the current pandemic, there were disturbing survey results indicating that the percentage of people rejecting vaccination was close to the percentage of people who would like to be vaccinated. This foreshadowed the difficulty of achieving the level of COVID-19 vaccination rates necessary to achieve herd immunity.

Our study adds to the existing literature by investigating the issue of consistency between the declared intention to vaccinate against COVID-19 and the actual behavior during the implementation of the vaccination program. Nearly all individuals who declared the willingness to vaccinate (96%) actually got vaccinated, with only six individuals becoming anti-vaccine and eight undecided. Much less consistency between declaration and behavior occurred in the previously anti-vaccine group of individuals, as follows: 20% of them became undecided, and 20% got vaccinated. The lowest support for mandatory vaccination was observed in the consistently anti-vaccine group, which indicates that their intention and consistent rejection of vaccination was rooted in a general negative attitude toward vaccination. The biggest positive change occurred in the previously undecided group, as follows: 56% of this group got vaccinated and only 11% became anti-vaccine. In other words, while there was a shift towards the decision to take COVID-19 vaccine, a minority continued to be opposed to a vaccine. A similar pattern of changes in attitudes (or behavior) towards vaccination was also observed in other countries (e.g., [6]). It is possible that the decision to vaccinate (despite previous hesitation) was influenced by the extensive information campaign promoting vaccination that appeared in the Polish media, as well as the benefits for vaccinated persons (e.g., possibility to travel abroad without COVID-19 testing). For those individuals who chose to reject vaccination or remained undecided, there was a significant decrease in support for mandatory vaccination, both against COVID-19 and general vaccination of children. Two mechanisms are possible here. On the one hand, the general change in attitudes toward vaccination to a more negative one may have caused these individuals to reject vaccination against COVID-19 (e.g., due to new arguments raised by anti-vaccine groups concerning the safety of the quickly developed COVID-19 vaccines). On the other hand, their behavior (non-vaccination) may have influenced attitudes toward mandatory vaccination as part of dissonance reduction.

There were no significant changes in attitudes toward mandatory vaccination of children between the first and second measurements in those who consistently opposed vaccination and those who consistently supported vaccination. This means that their declarations and then behaviors during the vaccination program were most likely rooted in attitudes, which remained unchanged.

We were also interested in what justifications stand behind the expressed intention and the actual behavior. The most important reasons to vaccinate against COVID-19 were “Increased safety of relatives and friends”, “Helping stop the pandemic”, “Vaccines are the most effective way of preventing diseases” and “Faster return to normal life”, This is consistent with the assumption that vaccine advocates base their decision on majoritarian common sense, concern for the common good (i.e., health), and respect for scientific authority; acceptance of vaccinations may result from a positive attitude towards science.

Typical arguments of vaccine opponents include risks associated with vaccine side effects, questioning the effectiveness of vaccines in preventing disease, and questioning the quality of vaccine research. The results of our study confirm this—the most common arguments against vaccination in both measurements were “Fear of side effects” and “Too little research”, followed closely by “I’m not sure the vaccine is safe and effective”. It seems that these arguments were raised especially often in regard to the COVID-19 vaccine, which may have led to the low vaccination rates compared to other established vaccines.

The presented research shows that declared anti-vaccine and hesitant attitudes are not set in stone, and even anti-vaccine individuals may become vaccinated under some circumstances. The large changes between declaration and behavior of the undecided group indicate that this group should especially be targeted by informational campaigns and means of persuasion toward vaccination, before anti-vaccine arguments sway them toward vaccine rejection. The rather obvious notion that vaccination attitudes change over time also means that they can be changed via targeted action, and knowing which arguments are the most important for pro-vaccine and anti-vaccine individuals may serve as basis for such action—debunking fears associated with side effects of vaccines and showing the quality and extent of research, as well as supporting the beliefs that vaccination is effective and protects not only particular individuals, but also their close ones. While our study identified some arguments important for supporters and rejecters of COVID-19 vaccination, this set of arguments is most likely incomplete, and further research is needed in this regard. Additionally, seeing that declared intent to vaccinate is not the same as actual behavior, more research should be based on identifying already vaccinated and strongly vaccine-rejecting groups (rather than just declarations of willingness), to truly investigate the causes of COVID-19 vaccine hesitancy.

## 5. Conclusions

The purpose of our study was to examine the relationship between attitudes toward COVID-19 vaccination (declared at the beginning of the national vaccination program) and actual behavior (investigated after six months). The attitudes of those declaring their intention to vaccinate appeared to be the best predictor of behavior—almost all of them got vaccinated. In the undecided group, more than half eventually decided to receive the vaccine, while among those declaring they would not vaccinate, the majority did not receive the vaccine within the next six months. These results may indicate that in the case of individuals with negative attitudes towards vaccination the arguments used in information campaigns promoting vaccination were not convincing. Thus, in-depth research should be conducted to understand their barriers and concerns about the COVID-19 vaccine and to design a persuasive strategy that is effective for them.

## Figures and Tables

**Table 1:** Comparison between declared vaccination intent at Time 1 and action at Time 2.

			Action at Time 2		
		Total	Anti-Vaccine	Undecided	Vaccinated
Declaration at Time 1	Anti-vaccine	217	126 (58%)	43 (20%)	48 (22%)
	Undecided	265	30 (11%)	86 (32%)	149 (56%)
	Pro-vaccine	393	6 (2%)	8 (2%)	379 (96%)
Total		875	162 (18%)	137 (16%)	576 (66%)

**Table 2:** Changes in reasons to not get vaccinated between Time 1 and Time 2 in the consistently anti-vaccine and undecided samples.

	Time 1		Time 2	
Reason	M	SE	M	SE
Do not believe coronavirus exists	2.621	0.160	3.895	0.186
Do not believe coronavirus is dangerous	3.211	0.161	5.018	0.176
Afraid of side-effects of vaccine	6.249	0.139	8.147	0.146
Had COVID, not afraid of getting infected again	1.989	0.147	4.007	0.202
Do not believe companies producing the vaccine are honest	5.540	0.152	7.484	0.158
Low probability that I will catch COVID	3.460	0.155	5.533	0.165
Too little clinical research conducted	6.295	0.137	8.316	0.136
Not sure if vaccine is safe and effective	6.509	0.130	8.281	0.142

**Table 3:** Changes in reasons to vaccinate between Time 1 and Time 2 n consistently pro-vaccine individuals.

	Time 1		Time 2	
Reason	M	SE	M	SE
Protecting self against COVID	8.625	0.094	6.720	0.088
Increased safety of relatives and friends	8.887	0.086	7.039	0.080
Helping stop the pandemic	8.781	0.085	6.794	0.086
Vaccine is free and voluntary	7.915	0.130	6.949	0.088
Inner peace from being safe	8.548	0.099	6.995	0.077
Vaccines are safe, controlled by medicines agencies	7.859	0.108	6.350	0.095
Vaccines are the most effective way of preventing diseases	8.730	0.089	7.064	0.067
Faster return to normal life	8.851	0.081	6.841	0.086

**Table 4:** Support for reasons to vaccinate between previously pro-vaccine, anti-vaccine and undecided groups who got vaccinated.

Intention to Behavior	Pro-Vaccine → Vaccinated		Undecided → Vaccinated		Anti-Vaccine → Vaccinated	
Reason	M	SE	M	SE	M	SE
Protecting self against COVID	6.760	0.102	4.946	0.163	3.458	0.287
Increased safety of relatives and friends	7.069	0.096	5.356	0.153	4.021	0.269
Helping stop the pandemic	6.823	0.100	4.953	0.160	3.375	0.281
Vaccine is free and voluntary	6.963	0.104	5.557	0.166	4.375	0.293
Inner peace from being safe	7.011	0.095	5.289	0.152	3.667	0.268
Vaccines are safe, controlled by medicines agencies	6.391	0.106	4.557	0.168	2.750	0.297
Vaccines are the most effective way of preventing diseases	7.079	0.091	5.141	0.145	3.667	0.256
Faster return to normal life	6.858	0.098	5.550	0.156	4.688	0.276
Protecting self against COVID	6.760	0.102	4.946	0.163	3.458	0.287

## Data Availability

The data presented in this study are openly available in OSF repository: https://osf.io/52wng/?view_only=970acc57db2e43cbb1e72f435d462826 (accessed on 15 December 2021).
